# Clinical and metabolic characteristics of treated hyperlipidemic patients additionally affected by subclinical hyperglycemia

**DOI:** 10.1186/s12944-016-0180-0

**Published:** 2016-01-13

**Authors:** Michael Leutner, Christian Göbl, Alice Wielandner, Eleonora Howorka, Marlies Prünner, Latife Bozkurt, Oliver Schlager, Silvia Charwat-Resl, Alexandra Kautzky-Willer

**Affiliations:** Department of Internal Medicine III, Division of Endocrinology and Metabolism, Unit of Gender Medicine, Medical University of Vienna, Waehringer Guertel 18-20, A-1090 Vienna, Austria; Department of Gynecology and Obstetrics, Division of Obstetrics and Feto-Maternal Medicine, Medical University of Vienna, Waehringer Guertel 18-20, A-1090 Vienna, Austria; Department of Biomedical Imaging and Image Guided Therapy, Medical University of Vienna, Waehringer Guertel 18-20, A-1090 Vienna, Austria; Department of Internal Medicine II, Division of Angiology, Medical University of Vienna, Waehringer Guertel 18-20, A-1090 Vienna, Austria

**Keywords:** Dyslipidemia, Prediabetes, Normal glucose regulation, Intima media thickness, Metabolic characteristics

## Abstract

**Background:**

Impaired glucose regulation (IGR) and hyperlipidemia (HL) are associated with an increased risk of developing a cardiovascular disease. Hyperlipidemic patients were shown to bear a greater risk for an increased intima media thickness (IMT). However little is known about differences between treated hyperlipidemic patients (HL) with normal (NGR) or impaired (IGR) glucose regulation.

**Methods:**

We performed a cross-sectional study, involving 96 non-diabetic HL patients with IGR (fasting plasma glucose of ≥ 100 mg/dl and <126 mg/dl or/and HbA1c-level of ≥ 5.7 and <6.5 %) or with NGR (HbA1c-level of <5.7 % and a fasting glucose <100 mg/dl). We compared metabolic characteristics and the IMT between the two groups. Insulin sensitivity in fasting conditions was described by HOMA-IR and QUICKI.

**Results:**

HL-IGR patients were older (57.6 ± 10.4 vs. 49.1 ± 8.7, *p* < 0.001), had higher carotid IMT measurements (IMT average: 0.68 ± 0.14 vs. 0.60 ± 0.09, *p* = 0.002; IMT right: 0.67 ± 0.15 vs. 0.60 ± 0.10, *p* = 0.013; IMT left: 0.63 vs. 0.57, *p* = 0.009), as well as a higher chance to exceed a cut-off value of ≥0.8 mm or insignificant stenosis within this investigation (OR: 3.9, 95 % CI: 1.15-13.22, *p* = 0.029) compared to HL-NGR-patients. Furthermore HL-IGR patients were characterised by a higher waist circumference (100.6 ± 10.1 vs. 91.6 ± 13.3, *p* < 0.001), higher fasting plasma glucose-levels (100.1 ± 10.8 vs. 88.1 ± 6.6, *p* < 0.001), higher HbA1c concentrations (5.8 ± 0.33 vs. 5.3 ± 0.24, *p* < 0.001) and C-peptide levels (2.70 vs. 2.10, *p* = 0.012). Age and CVD status were in general the only two variables which independently explained IMT.

**Conclusion:**

Our study showed that among patients with treated hyperlipidemia the presence of IGR characterised subjects who were older and had a significantly higher risk for an increased IMT compared with those maintaining NGR. Further studies are necessary to evaluate if this specific subpopulation with IGR can benefit from a more strict multifactorial management and perhaps from an additional early antihyperglycaemic treatment.

## Background

Dyslipidemia is a known risk factor for an increased carotid intima media thickness [1] and for the development of atherosclerosis [2]. There is a positive correlation between blood lipid levels and cardiovascular risk [3, 4], so antilipidemic treatment is a cornerstone of the prevention of cardiovascular disease [5].

In addition to dyslipidemia, prediabetes can also be a risk factor for the development of cardiovascular disease [6] and can also associate with increasing IMT-values [7].

While studies show that dyslipidemia can increase the chance of developing prediabetes [8, 9], the “Paris Prospective study” showed that the combination of prediabetes- or diabetes with high blood lipid values indeed is a major risk factor for coronary heart disease [10]. However, initiation of pharmacotherapy in prediabetic patients is nowadays controversial, partly because there are no data available that show whether the specific population of treated hyperlipidemic patients with a subclinical diabetes (HL-IGR) can benefit from an additional antihyperglycemic treatment.

In summary, while there is a body of evidence that both dyslipidemia and prediabetes associate with increased cardiovascular risk, little is known yet about both the metabolic and clinical characteristics of the specific subpopulation of treated hyperlipidemic patients with an impaired glucose regulation (HL-IGR) and the optimal adjustment of lipid- and glucose values in these patients.

Therefore, the objective of this study is to compare the clinical- and metabolic characteristics as well as the carotid intima-media thickness in patients with a treated hyperlipidemia additionally affected by prediabetes.

## Methods

### Study participants and experimental procedures

A cross-sectional study, prospectively involving 96 patients undergoing hyperlipidemia therapy was conducted between August 2011 and May 2013. The study was approved by the Ethics Committee of the Medical University of Vienna (EC No.: 542/2011) and performed in accordance with the Declaration of Helsinki. All participants gave written informed consent.

Patients were recruited from the outpatient department of the division of Endocrinology and Metabolism of the Medical University of Vienna.

To be included in this study, participants had to be aged between 35 and 75 years and had to have a stable dyslipidemic treatment according to the ESC/EAS guidelines [11] which hadn’t changed in the last three months before entering the study. Exclusion criteria was either diagnosed diabetes mellitus type 1 or manifest diabetes mellitus type 2 (fasting plasma glucose ≥126 mg/dl or HbA1c ≥ 6.5 % or antihyperglycemic treatment), metabolic disorders (such as diagnosed hereditary dyslipidemia), liver diseases other than non-alcoholic fatty liver disease, e.g. viral hepatitis, HIV, anti-hormonal therapy in men (e.g. to treat prostate cancer), corticosteroid therapy within eight weeks prior to study commencement, immune suppression, chronic renal insufficiency, hepatotoxic medication and alcohol abuse. Participants were divided into two groups: one group included 48 treated hyperlipidemic patients with impaired glucose regulation (HL-IGR) and a high risk of developing diabetes (fasting plasma glucose ≥100 mg/dL and <126 mg/dL or HbA1c ≥5.7 and <6.5 %) according to the guidelines of the American Diabetes association [12]. The other group included 48 patients with a diagnosed and treated hyperlipidemia with a normal glucose regulation with Hba1c-levels <5.7 % and fasting glucose levels <100 mg/dl (HL-NGR). Participants’ general medical history and smoking habits were assessed by questionnaire. Anthropometric data included body weight, height and waist circumference, which were measured according to standardized measurements as previously used in other studies [13]. Blood pressure and heart frequency were measured on the right arm after a five minute resting period in a sitting position.

A positive cardiovascular history was recorded if one of the following existed: history of stroke, peripheral artery disease, coronary heart disease, myocardial infarction and angina pectoris.

Insulin resistance (or sensitivity) in fasting conditions was assessed by HOMA-IR [14] and QUICKI [15]. We used a cut-off point of ≥0.8 mm for defining the start of an insignificant atherosclerosis in the carotis-IMT as previously done in the study of Riccioni et al. [2]

### Ultrasound measurements

IMT measurements were performed using high-resolution B-mode ultrasonography (Acuson XP 128, Siemens Medical Solutions, USA) using a 9 MHz linear transducer probe. Measurements were performed in the distal common carotid artery, 1.5–2 cm proximal to the carotid bifurcation on the far vessel wall. The IMT was defined as the distance between the leading edge of the lumen-intima echo and the leading edge of the media-adventitia echo. Frozen end-diastolic images of the far wall were recorded in a longitudinal view of the artery and three IMT measurements were obtained from each common carotid artery. For further analyses the average value of these three measurements was calculated.

All measurements were performed by an experienced co-investigator (Silvia Charwat-Resl) under supervision of another co-investigator (Oliver Schlager) blinded to any additional clinical information.

### Laboratory methods

Blood samples were taken after an at least ≥10 h overnight fasting period. Laboratory tests measured insulin, C-peptide, fasting plasma glucose, lipid (triglycerides, HDL, total cholesterol) levels and pro-BNP using international standard laboratory methods at the certified Department of Medical and Chemical Laboratory Diagnostics (http://www.kimcl.at/). LDL was calculated with the Friedewald-formula in persons with <400 mg/dl [16].

### Statistical analysis

Continuous variables were summarized by means ± standard deviations (SD), categorical variables by counts and percentages as not otherwise indicated. For variables, which weren’t normally distributed, median and interquartile range were stated. Normal distribution of continous variables was verified by the Shapiro-Wilk-test. The following four variables were transformed using logarithmic transformation (ln[x]): triglycerides, pro-BNP, Lipoprotein-a and hsCRP as they were strongly right-skewed with the lower limit of zero. Group based comparisons (i.e. normal glucose tolerance “NGT” vs. impaired glucose regulation “IGR”) were compared by using student’s *t*-test (or by the Wilcoxon rank sum test if normality assumption was violated) and Fisher’s exact test, respectively. The associations between continuous variables were assessed by Pearson’s product moment correlation. Linear (for continuous outcomes) and linear regression models were used for multivariable adjustment (e.g. age and sex). 

**Fig. 1:**
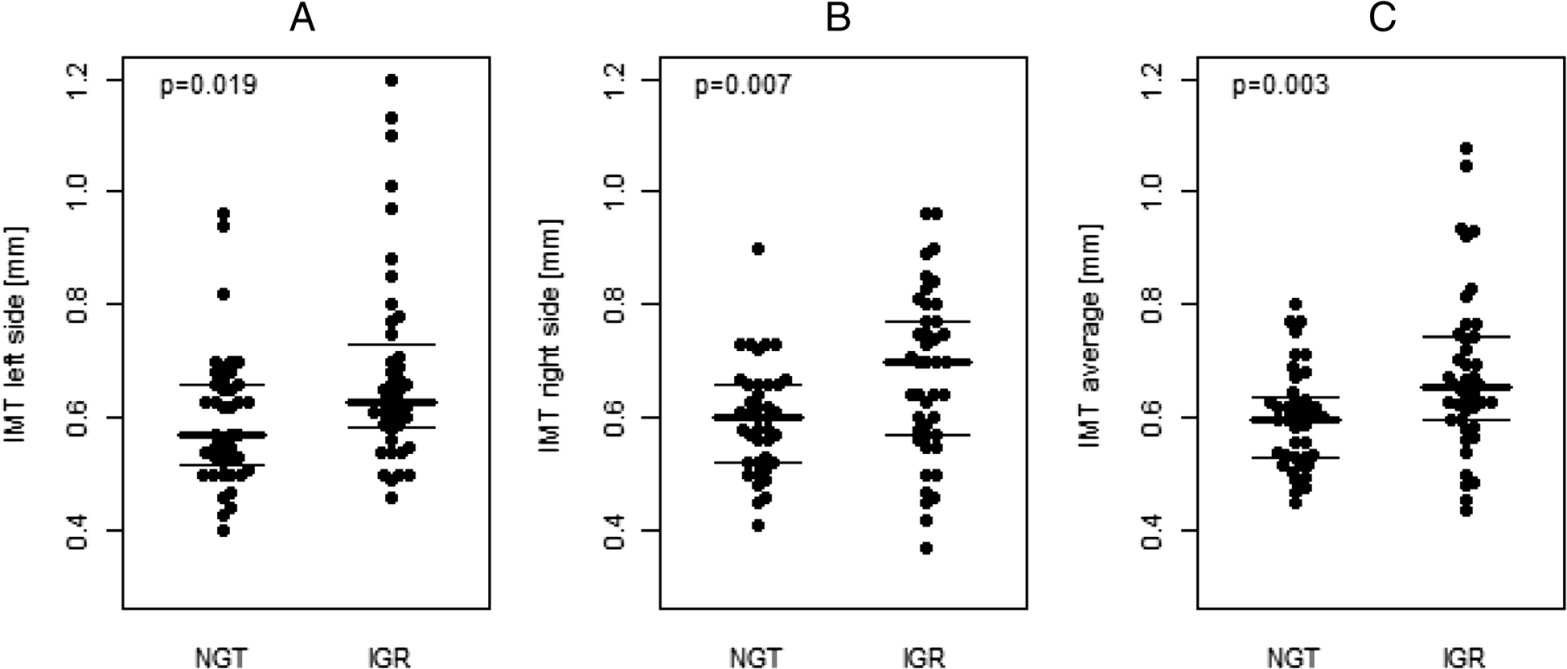
Comparison of IMT measurements. Bee swarm plot of carotid IMT data in normal glucose tolerant hyperlipidemic patients (NGT) vs. hyperlipidemic patients affected by impaired glucose regulation (IGR): left side (**a**), right side (**b**), average (**c**). Lines indicate first, second (median) and third quartiles

Statistical analysis was performed with SPSS (V20). Only bee swarm plots (Fig. 1) were constructed with R (V3.1.1) and contributed packages [17]. A two-sided *p*-value ≤0.05 was considered statistically significant. *P*-values were interpreted descriptively and there were no considerations to adjust for multiplicity in this observational study.

## Results

**Fig. 2:**
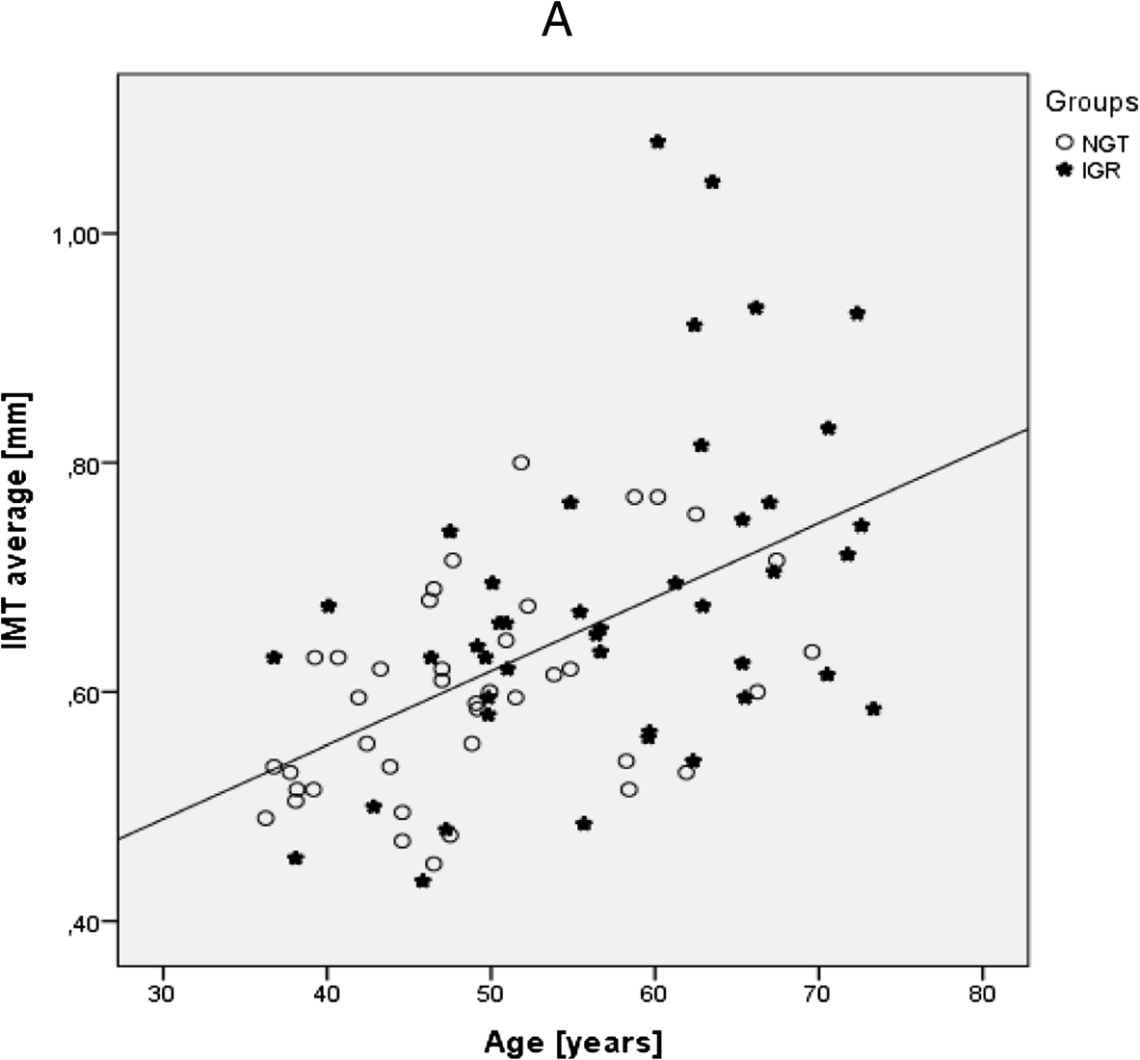
Association between average carotid IMT measurements and age (**a**)

**Fig. 3:**
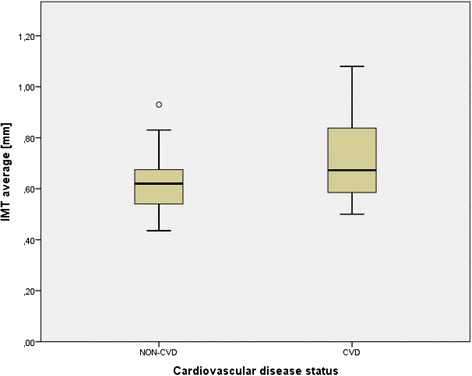
Box-plot for IMT average grouped by cardiovascular disease status

In the total study population of *n* = 96 patients with hyperlipidemia, *n* = 26 (27,1 %) showed impaired fasting glucose (FPG ≥ 100 mg/dl), *n* = 36 (37,5 %) showed elevated HbA1c (≥5.7 %). 48 (50 %) subjects were positive of at least one of both criteria for prediabetes (HL-IGR) and 48 (50 %) subjects weren’t positive of any criteria for prediabetes and formed the HL-NGR group. A descriptive comparison of study participants (HL-IGR vs. HL-NGR) is provided in Table 1 and shows, that hyperlipidemic patients additionally affected by subclinical hyperglycemia were older, showed a higher degree of central obesity (i.e. waist circumference) and significantly elevated parameters of carbohydrate metabolism (i.e. HbA1c, glucose, and C-peptide concentrations) as compared to normoglycemic hyperlipidemic subjects. Moreover, univariable comparisons indicated significantly higher carotid IMT measurements in the subgroup with borderline hyperglycemia (Fig. 1), as well as a higher chance to exceed a cut-off value of ≥0.8 mm or insignificant stenosis within this investigation (OR: 3.9, 95 % CI: 1.15–13.22, *p* = 0.029). An analysis of continuous data revealed that age (*r* = 0.532, *p* < 0.001) is related to average IMT measures (Fig. 2). There was also a weak positive correlation of fasting plasma glucose and IMT-values (*r* = 0.237, *p* = 0.029), however this correlation lost significance when the four patients with IMT values >0.9 mm were excluded from the statistical analysis. No association with IMT was observed for lipid levels (total cholesterol (TC): *r* = -0.04, *p* = 0.693; LDL-cholesterol: *r* =−0.08, *p* = 0.455; HDL-cholesterol: *r* = -0.095, *p* = 0.395; ln(Tg): *r* = 0.17, *p* = 0.108), pack years of smoking (*r* = 0.16, *p* = 0.188), parameters of body composition (BMI: *r* =−0.06, *p* = 0.683; waist circumference: *r* = 0.09, *p* = 0.446) or insulin sensitivity (QUICKI: *r* =−0.12, *p* = 0.313; HOMA: *r* = 0.18, *p* = 0.137). The correlation between waist circumference and HOMA-IR (*p* < 0.001), as well as the correlation between waist circumference and QUICKI-test (*p* = 0.001) was significant. Additional calculations in a subgroup with a history of CVD showed significantly higher IMT levels as compared to those without CVD (IMT average: 0.72 ± 0.18 vs. 0.62 ± 0.10, *p* = 0.043; right carotid intima media thickness “right IMT”: 0.7 ± 0.15 vs. 0.62 ± 0.12, *p* = 0.035; left carotid intima media thickness “left IMT”: 0.74 ± 0.24 vs. 0.61 ± 0.12, *p* = 0.098;) as visualized in Fig. 3. Multivariable models were performed to adjust for potential confounding effects of age, sex, glucose and waist circumference: Age and CVD status were the only variables which independently explained IMT. The statistical correlation between CVD status and HL-IGR or HL-NGR was analyzed using the Chi-squared-test and wasn’t significant (*p* = 0.163). After the exclusion of patients with a history of CV-events, HL-IGR patients still had significantly higher carotid IMT values compared with HL-NGR patients (*p* = 0.044). There was an insignificant difference between the mean duration of the dyslipidemic treatment in the HL-NGR patients compared with the HL-IGR patients (774 days vs. 1418.50 days; *p* = 0.199). There was also an insignificant difference, which is described in Table 1, in the type and the medication dose of dyslipidemic treatment received by the two groups. Additional statistical analyses showed that there isn’t a significant difference between the different types of statins regarding glucose-levels (*p* = 0.481) and HbA1c-levels (*p* = 0.511).

**Table 1:** Characteristics of the study sample

	n (NGT/IGR)	NGT	IGR	*p*-value
Age [years]^a^	48/48	49.1 ± 8.7	57.6 ± 10.4	<0.001
sex [m]^b^	48/48	33 (68.8)	29 (60.4)	0.522
BMI [kg/m^2^]^a^	45/47	27 ± 4.4	28.4 ± 4.1	0.102
Waist [cm]^a^	41/45	91.6 ± 13.3	100.6 ± 10.1	<0.001
SBP [mmHg]^a^	32/34	127.2 ± 10.7	132.7 ± 18.2	0.132
DBP [mmHg]^a^	32/34	80.8 ± 8.8	80.1 ± 11.2	0.764
TC [mg/dl]^c^	48/48	214.5 (181.5–273.0)	217.5 (183.5–272.8)	0.980
LDL-C [mg/dl]^c^	46/45	116.4 (95.8–168.5)	120.0 (98.0–169.1)	0.730
HDL-C [mg/dl]^c^	46/44	45.5 (38.5–63.0)	49.0 (38.3–61.0)	0.878
NHDL-C [mg/dl]^c^	46/44	159.0 (130.5–204.8)	158.0 (134.0–207.5)	0.881
ln(TG)^a^	48/48	5.3 ± 0.8	5.4 ± 0.7	0.380
ln(Lip.a)^c^	42/43	2,89 (2.30–3.29)	3.47 (2.30–4.34)	0.052
IMT-left [mm]^c^	43/43	0.57 (0.51–0.66)	0.63 (0.58–0.75)	0.009
IMT-right [mm]^a^	40/43	0.60 ± 0.10	0.67 ± 0.15	0.013
IMT-av [mm]^a^	40/43	0.60 ± 0.09	0.68 ± 0.14	0.002
Glucose [mg/dl]^a^	48/46	88.1 ± 6.6	100.1 ± 10.8	<0.001
Insulin [μU/ml]^a^	46/44	8.98 ± 5.6	12.1 ± 9.7	0.069
C-Pept [ng/ml]^c^	38/39	2.10 (1.68–2.85)	2.70 (2.30–3.80)	0.012
HbA1c [%]^a^	48/47	5.3 ± 0.24	5.8 ± 0.33	<0.001
HOMA-IR^a^	39/39	1.95 ± 1.3	2.5 ± 1.8	0.120
QUICKI^c^	39/39	0.35 (0.33–0.40)	0.34 (0.31–0.38)	0.248
ln(proBNP) [pg/ml]^c^	43/39	3.87 (3.31–4.41)	4.14 (3.34–4.73)	0.370
ln(hsCRP) [mg/d]^a^	45/45	−1.97 ± 1.26	−1.90 ± 0.97	0.764
CVD^b^	46/45	6 (13.0)	11 (24.4)	0.188
Mean steps/7d^c^	38/38	6969 (5904–9092)	7707 (6145–9860)	0.747
CRP [mg/dl]^c^	45/45	0.14 (0.09–0.40)	0.16 (0.09–0.34)	0.904
smoking [PY]^c^	36/34	15.0 (0.0–27.3)	5.5 (0.0–27.8)	0.511
Bezafibrate [dd (mg)]^c^	14/10	400 (400–400)	400 (400–400)	0.379
Rosuvastatin [dd (mg)]^c^	15/23	10 (10–20)	20 (10–20)	0.394
Atorvastatin [dd (mg)]^c^	4/4	60 (32.5–80)	30 (17.5–50)	0.743
Simvastatin [dd (mg)]^c^	5/8	20 (20–20)	20 (20–80)	0.314
Ezetimibe [n(%)]^b^	4/5	4 (9.3)	5 (11.9)	0.697
Nicotinic Acid [n(%)]^b^	3/2	3 (7.0)	2 (4.8)	0.664
Mean treatment duration of the last taken dyslipidemic medication (days)^c^	46/46	774 (338.50–2331.25)	1418.50 (512.75–3168.50)	0.199

^a^t-test

^b^chi-square test

^c^Wilcoxon rank-sum test

Data are number of observarion (n) and means ± standard deviation

*BMI* body mass index, *SBP* systolic blood pressure, *DBP* diastolic blood pressure, *TC* total cholesterol, *LDL-C* low density lipoprotein cholesterol, *HDL-C* high density lipiprotein cholesterol, *NHDL-C* non high density lipiprotein cholesterol, *TG* triglycerides, *Lip.a* lipoprotein (a), *IMT* carotid intima media thickness, *IMT av* IMT average, *C-Pept* C-Peptide, *HbA1c* glycated haemoglobin A1c, *HOMA-IR* homeostatic model assessment of insulin resistance, *QUICKI* quantitative insulin sensitivity check index, *hsCRP* high-sensitivity C-reactive protein, *CVD* cardiovascular disease, *7d* 7 days, *PY* pack years, *dd* daily dose

## Discussion

This prospective cross-sectional study investigates the differences in treated HL-IGR-patients and HL-NGR-patients, based upon metabolic characteristics and the IMT.

The main findings in our study were the significantly higher measurements of the IMT in HL-IGR patients, as well as a higher occurrence of a subclinical carotid-atherosclerosis, which was defined as a cut-off value of ≥0,8 mm [2]. HL-IGR patients also showed a significantly higher degree of central obesity, especially in the waist circumference compared to HL-NGR patients. In addition we observed an association between the IMT and age in treated dyslipidemic patients.

However literature about hyperlipidemic patients with screen-detected impaired glucose regulation especially regarding atherosclerosis is sparse. In general it is known that hyperlipidemia and hyperglycemia [18–21] are both risk-factors of atherosclerosis on their own, but there are no studies which have investigated the combined effect of these two factors. In our study HL-IGR patients have a higher IMT than HL-NGR patients and also more often an insignificant stenosis which could indicate a cumulative effect. However our study groups had an imbalanced mean age and it is well known that age is also a predictor of atherosclerosis [18]. Nevertheless we think that especially in elderly hyperlipidemic patients, the screening for IGR may be important, not only concerning the higher risk of atherosclerosis but also other complications. To clarify this issue it is necessary to investigate in larger studies whether the specific population of HL-IGR patients could benefit from an additional antihyperglycemic treatment in addition to the antilipidemic treatment in order to prevent the progression of atherosclerosis.

Another interesting but yet unresolved issue is the potential association between fasting plasma glucose and IMT in hyperlipidemic patients. The currently existing data is controversial regarding this topic [18, 22, 23]. A relationship could also be based on shared risk factors and nonglucose pathways [24, 25].

On the other hand our study results confirm the existing data and show that there is an independent positive correlation of age and the IMT [26, 27]. This results point out that antilipidemic treatment cannot curb this correlation.

In contrast to the study of Salonen et al. [26] we were unable to find a correlation between smoking and the progression of atherosclerosis in the IMT of treated dyslipidemic patients. Our finding that HL-IGR patients have a significantly higher degree of central obesity, especially in the waist circumference confirms the close relationship between abdominal Obesity and Prediabetes [28]. Additional statistical analyses confirmed this assumption and showed that there is a significant correlation between waist circumference and insulin resistance.

HL-IGR patients in our study have higher insulin values compared with HL-NGR-patients reflecting more pronounced insulin resistance. Indeed HL-IGR patients have a greater tendency for insulin resistance calculated by the HOMA-formula compared to HL-NGR patients. In the study of Kostapanos et al., the investigators showed that longtime statin-dosage can aggravate insulin-resistance in hyperlipidemic-patients with impaired fasting glucose [29] and our results point out that HL-IGR patients had more often and for a longer duration an antilipidemic statin-treatment, compared to HL-NGR patients and also a significantly higher waist circumference which is a marker of insulin resistance [28]. However we couldn’t found significant differences between the different types of statin-treatment regarding glucose-levels and HbA1c-levels. Nevertheless there is also controversial data available which show that statins don’t have a diabetogenic effect [30, 31]. HL-IGR-patients in our study have significantly higher fasting-glucose-levels and thus also the impact of statin therapy on glucose metabolism has to be considered. Prediabetes is also a factor associated with mortality as shown in the Decode study [32].

Furthermore the sub-analysis that hyperlipidemic patients in our study, who had a cardiovascular event in the past have significantly higher IMT-measurements compared to those without and that CVD-status in treated hyperlipidemic patients is an independent variable which explains the IMT, confirms existing studies and is in line with a meta-analysis of Lorenz et al., who also showed that the IMT is directly associated with the occurence of cardiovascular events [33].

Nevertheless additional statistical analyses of our data showed that after the exclusion of patients with a history of cardiovascular events, HL-IGR patients still had significantly higher carotid IMT measures compared with HL-NGR patients (*p* = 0.044). This data shows that there is a cumulative effect of dyslipidemia and IGR which has been rarely examined up to now.

In our study there was no correlation between the lipid levels (LDL-cholesterol, TC, HDL-cholesterol) and the carotid intima media thickness. This lack of association could be explained by the fact that all of the study participants had lipid-lowering treatment when they entered the study. Nevertheless it would be necessary to prove in larger studies if HL-IGR patients can benefit of a more strict and exact antilipidemic treatment, as previously shown in the JART study in which patients with a strictly treated LDL-cholesterol level of >100 mg/dl and <120 mg/dl for one year showed a reduction of atherosclerosis by 4.29 % (0.040 mm) [34] as well as to prove if HL-IGR patients can benefit of an additional antihyperglycaemic treatment in order to decrease the risk for the progression of atherosclerosis.

A suggestion of the authors of this study regarding antihyperglycaemic treatment in HL-IGR patients is to encourage life-style modification with an improvement in nutrition and increase of physical activity according to the Austrian Diabetes Association guidelines [35]. Physical activity should be increased to 30 min per day or a minimum of 150 min per week. In addition, as recommended by the American Diabetes Association, early oral antihyperglycaemic drug therapy with metformin could be helpful in the high-risk population of HL-IGR-patients in order to decrease the risk of comorbidities, especially for atherosclerosis [36]. The recently presented EMPA-REG study is the first study which shows that diabetic patients who received the SGLT-2 inhibitor empagliflozin had a significantly lower risk of death of cardiovascular events compared with a placebo group [37]. Research indicates that empagliflozin in addition to conservative treatment could be even more useful in HL-IGR- patients than metformin, especially regarding the high risk of atherosclerosis in this specific population. However more research needs to be done because no studies investigated the potential impact of empagliflozin in hyperlipidemic patients additionally affected by prediabetes up to now.

Our study has some limitations: The most important limitation of our study is the differences in the mean age between the two study groups. In addition to the small number of participants our results are restricted due to the definition of IGT by using fasting blood samples and HbA1c and not the oral glucose tolerance test (OGTT) [38]. In addition some data from our study groups was missing, and the two groups received different treatment modalities.

## Conclusion

We summarize, that HL-IGR-patients in comparison to HL-NGR-patients are characterised as being older, having a higher risk of atherosclerosis and may need in addition to an optimal antilipidemic- also an antihyperglycemic treatment to prevent cardiovascular disease and progressive atherosclerosis. Prospective studies with a larger number of participants and different age-groups would be necessary in order to get more conclusive results. A recommendation of our study is that HL-IGR-patients need more frequent investigations (e.g. blood tests, IMT measurement) and that the medication should be adjusted at regular follow up-appointments.
